# Resource partitioning of phytoplankton metabolites that support bacterial heterotrophy

**DOI:** 10.1038/s41396-020-00811-y

**Published:** 2020-10-23

**Authors:** Frank Xavier Ferrer-González, Brittany Widner, Nicole R. Holderman, John Glushka, Arthur S. Edison, Elizabeth B. Kujawinski, Mary Ann Moran

**Affiliations:** 1 Department of Marine Sciences, University of Georgia, Athens, GA 30602 USA; 2 Department of Marine Chemistry and Geochemistry, Woods Hole Oceanographic Institution, Woods Hole, MA 02543 USA; 3 Department of Biochemistry and Complex Carbohydrate Research Center, University of Georgia, Athens, GA 30602 USA

**Keywords:** Microbial ecology, Bacteria

## Abstract

The communities of bacteria that assemble around marine microphytoplankton are predictably dominated by Rhodobacterales, Flavobacteriales, and families within the Gammaproteobacteria. Yet whether this consistent ecological pattern reflects the result of resource-based niche partitioning or resource competition requires better knowledge of the metabolites linking microbial autotrophs and heterotrophs in the surface ocean. We characterized molecules targeted for uptake by three heterotrophic bacteria individually co-cultured with a marine diatom using two strategies that vetted the exometabolite pool for biological relevance by means of bacterial activity assays: expression of diagnostic genes and net drawdown of exometabolites, the latter detected with mass spectrometry and nuclear magnetic resonance using novel sample preparation approaches. Of the more than 36 organic molecules with evidence of bacterial uptake, 53% contained nitrogen (including nucleosides and amino acids), 11% were organic sulfur compounds (including dihydroxypropanesulfonate and dimethysulfoniopropionate), and 28% were components of polysaccharides (including chrysolaminarin, chitin, and alginate). Overlap in phytoplankton-derived metabolite use by bacteria in the absence of competition was low, and only guanosine, proline, and *N*-acetyl-d-glucosamine were predicted to be used by all three. Exometabolite uptake pattern points to a key role for ecological resource partitioning in the assembly marine bacterial communities transforming recent photosynthate.

## Introduction

The marine dissolved organic carbon (DOC) reservoir plays two critical roles in the global carbon cycle. The first is as a long-term repository of carbon nearly equal in magnitude to the inorganic carbon stored in the atmosphere. This role is fulfilled by ~660 Pg C of refractory organic compounds that accumulate in seawater, some with lifetimes of many thousands of years [1]. The second is as the primary source of substrates for heterotrophic marine microbes. This role is fulfilled by ~0.2 Pg of highly bioreactive organic compounds that are rapidly cycled by bacterioplankton [2], some with turnover times on the order of hours [3–5]. Recent chemical analysis of bulk marine DOC concentrated from seawater has shown it to be conservatively composed of tens of thousands of distinct organic compounds [6, 7]. Yet chemical analysis of the bioreactive subset of marine DOC has thus far been dominated by targeted analysis of a limited number of core metabolites, such as amino acids, sugars, and select biopolymers [8–11]. Untargeted chemical approaches have lagged behind, plagued by low concentrations, similar physical properties of salt and polar metabolites, and short lifetimes of component metabolites. Thus many critical but largely invisible chemical connections between marine microbes remain unstudied.

The primary source of labile metabolites for surface ocean bacteria is phytoplankton. It is estimated that these microbial autotrophs release 10–20% of net primary production (NPP) into the marine DOC pool in coastal systems [12] and up to 40% in oligotrophic systems [13], although this percentage varies across species and growth conditions [14, 15]. Mechanisms by which metabolites are released from phytoplankton cells range from passive leakage of small and uncharged molecules [16], to active exudation related to redox balance, defense, and signaling [17–19], to photosynthetic overflow due to stoichiometric imbalance [15]. Labile organic compounds also become available by phytoplankton cell death from processes such as viral lysis, protist grazing, and senescence [20–22]. Once metabolites are released or excreted from phytoplankton cells, heterotrophic bacteria play the dominant role in their transformation.

Three marine bacterial taxa have consistently been found associated with microphytoplankton in natural marine environments and phytoplankton cultures, and are thought to dominate processing of recently-fixed carbon using genes that allow them to quickly respond to transient nutrient pulses [23]. Members of these three groups, the Rhodobacterales, Gammaproteobacteria, and Flavobacteriales, appear to specialize on different components of bioreactive DOC [11, 23–25]. Rhodobacterales typically play a prominent role in processing low molecular weight (LMW) phytoplankton-derived metabolites [26, 27], Flavobacteriales largely transform high molecular weight carbohydrate polymers [28, 29], and several copiotrophic Gammaproteobacteria families utilize compounds from both classes [30, 31]. Here, we address the ecological basis of this widespread taxonomic pattern by generating metabolite uptake profiles for three model bacterial species, one from each of the major phytoplankton-associated groups, when growing on a microphytoplankton exometabolite pool in the absence of competition from other bacteria.

Pairwise co-culture systems were established with the diatom *Thalassiosira pseudonana* as the sole source of substrates for *Ruegeria pomeroyi* DSS-3 (Rhodobacterales), *Stenotrophomonas* sp. SKA14 (Xanthomonadales), or *Polaribacter dokdonensis* MED152 (Flavobacteriales). *T. pseudonana* was selected as the autotrophic member of the model systems because as a group, marine diatoms contribute ~20% of global NPP [32]. The heterotrophic bacterial strains were selected because they have high identity to 16S rRNA genes from phytoplankton cultures or flow-sorted phytoplankton cells, with percent similarities as high as 99.6% for *R. pomeroyi* [33–36], 98.8% for *Stenotrophomonas* sp. SKA14 [33], and 97.2% for *P. dokdonensis* [35, 37]. To overcome some challenges of direct chemical analysis of low-concentration compounds in seawater, we used two biological vetting approaches that highlighted metabolites most likely to be important in phytoplankton-bacteria carbon exchange. In the first, expression patterns of bacterial transporters and catabolic genes were used to identify the cellular machinery for carbon acquisition activated by each strain when growing in co-culture. In the second, drawdown of diatom-derived exometabolites from the co-culture media was used to distinguish compounds decreasing in the presence of bacteria. Metabolite concentrations were measured using liquid chromatography-mass spectrometry (LC-MS) and heteronuclear single quantum coherence (HSQC) nuclear magnetic resonance (NMR) spectroscopy. Both biological vetting strategies relied on bacterial activity to spotlight compounds within a complex pool of dilute metabolites that were likely supporting bacterial heterotrophy.

## Methods

### Co-cultures

Three bacterial strains were introduced individually into 7-day diatom co-cultures. Samples were collected after 8, 24, and/or 48 h and analyzed for bacterial response via transcriptomics (to measure regulation changes) or via mass spectrometry (MS) or NMR (to measure metabolite drawdown). To initiate co-cultures, *T. pseudonana* 1335 (National Center for Marine Algae) was grown axenically in organic carbon-free medium L1 +Si [38] in 1900-ml vented polystyrene tissue culture flasks at 18 °C under 16 h light at 160 µmol photons m^−2^ s^−1^ and 8 h dark. For samples used in NMR analysis, ^13^C bicarbonate was used to make the L1 medium. After the diatom cultures had been growing for 7 days, bacteria pre-grown in YTSS medium were washed five times in sterile L1 medium and inoculated into three replicate diatom cultures at ~10^**6**^ cells ml^−1^; three flasks remained uninoculated. Following incubation in the light for 8 h, *T. pseudonana* cells were removed by pre-filtration through 2.0-µm-pore-size filters, and bacteria were collected on 0.2-µm-pore-size filters. Filters were immediately flash frozen in liquid nitrogen and stored at −80 °C until processing and the filtrate was stored frozen for subsequent chemical analysis. Filtrate was also obtained 24 and 48 h after bacterial inoculation for chemical analysis. The control for transcriptome analysis was established by growing bacteria in a defined glucose medium (L1 medium +Si with 2.5 mM glucose); this masked signals of glucose utilization in the co-cultures but provided baseline transcriptomes of actively growing bacteria. Bacterial strains were similarly inoculated into the control medium, collected on 0.2 pore-size filters after 8 h, and flash frozen. Detailed methods are given in Supplementary File 1.

### Cell counts

Culture samples were fixed to a final concentration of 1% glutaraldehyde, incubated at 4°C for 20 min, and stored at −80 °C. Just prior to analysis, an internal standard of 5-μm fluorescent beads was added (Spherotech, Lake Forest, IL, USA), followed by staining for 15 min with SYBR Green I (final concentration 0.75X; Life Technologies, Waltham, MA, USA). Samples were analyzed on an Agilent Quanteon flow cytometer (Acea, Biosciences Inc., San Diego CA). Fluorescence was detected with a 405 nm laser using a 530/30 bandpass filter for SYBR Green (bacteria) and a 695/40 bandpass filter for chlorophyll a. There was no bacterial contamination of axenic cultures based on scattergrams from flow cytometry and aliquots from axenic cultures spread onto YTSS plates.

### RNA-seq analysis

Filters were incubated in SDS (0.6% final concentration) and proteinase K (120 ng μl^–1^ final concentration). RNA was extracted from duplicates of each treatment by adding an equal volume of acid phenol:chloroform:isoamyl-alcohol, followed by shaking, centrifugation, collection of the supernatant, and addition of an equal volume of chloroform:isoamyl-alcohol. RNA was recovered from the supernatant, treated to remove rRNA, and sequenced on an HiSeq Illumina 2500. Quality control of the 207 million 50-bp reads was performed using the FASTX toolkit. Reads aligning to rRNA were removed and remaining reads were mapped to the bacterial genomes. Genes with differential expression were determined with DESeq2 [39], and are available in Supplementary Tables S1, S2, and S3. The dbCAN web resource was used for identification of carbohydrate-active enzyme annotations [40]. Raw RNA-seq data are available in the NCBI SRA BioProject database under accession PRJNA448168. Detailed methods are given in Supplementary File 1. Microbial genome sequences are available at NCBI RefSeq under accession numbers ASM14940v2 (*T. pseudonana* CCMP1335), ASM1196v2 (*R. pomeroyi* DSS-3), ASM15857v1 (*Stenotrophomonas* sp. SKA14), and ASM15294v2 (*P. dokdonensis* MED152).

### Mass spectrometry analysis

Chemical analysis was conducted on filtered spent media from the co-cultures and axenic *T. pseudonana* culture using an uninoculated L1 as the medium blank. For MS analysis, 8, 24, and 48 h co-culture spent media were analyzed. Metabolites were derivatized with benzoyl chloride [41] by modification of methods from Oehlke et al. [42] and Wong et al. [43], extracted using a solid phase resin (Agilent, Bond Elut PPL), and analyzed using ultra high performance liquid chromatography coupled with electrospray ionization and tandem MS with modifications to Kido Soule et al. [44]. Metabolite peak areas were selected and integrated using Skyline [45, 46] (Fig. S1). MS metabolites were evaluated statistically in MATLAB by comparing adjusted sample concentrations grouped across time points using a one-way ANOVA (*α* = 0.05). Post hoc Dunnett’s test was used to compare each co-culture to the axenic *T. pseudonana* treatment, and *p* values were adjusted for multiple comparisons. Outliers were defined as values that exceeded three scaled median absolute deviations and were excluded from statistical analysis. All MS data are available at MetaboLights under accession number MTBLS1751. Detailed methods are given in Supplementary File 1.

### NMR analysis

For NMR analysis, 5 ml of 48 h co-culture spent medium was lyophilized, homogenized dry using 5 × 3.5 mm glass beads, reconstituted in dimethyl-sulfoxide-d6, and re-homogenized. The supernatant was analyzed using two-dimensional HSQC NMR (Bruker 800 MHz NEO with 1.7 mm cryoprobe) using acquisition parameters modified from a hsqcetgpsisp2.2 pulse program (TopSpin V4.0.6). The indirect ^13^C (f1) dimension had a spectral width of 90.0027 ppm, 128 data points, and a carrier frequency of 45 ppm. The direct ^1^H dimension (f2) had a spectral width of 13.0255 ppm, 4166 data points, and a carrier frequency of 3.691 ppm. Spectra were processed in MNOVA and transformed spectra were auto-phased, baseline-corrected, and referenced along f1 and f2 to DSS-d6 (0.0, 0.0 ppm). All peaks above noise were manually integrated (Fig. S2). A MATLAB workflow was used to normalize, scale, and analyze spectral features. *p* values were calculated for all peak integrals using the [ttest] function in MATLAB for sample pairs. False discovery rates and *q* values were calculated using MATLAB built-in function [mafdr]. Raw data, peak lists, and analysis scripts are available at MetaboLights under accession MTBLS1544. Detailed methods are given in Supplementary File 1.

## Results

**Fig. 1:**
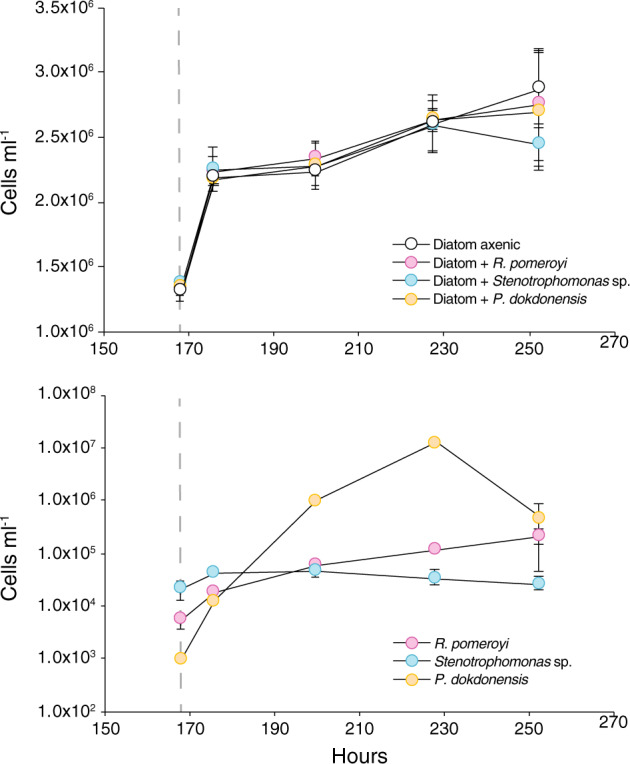
Cell numbers in co-culture experiments after inoculation of bacterial strains into *T. pseudonana* cultures at 168 h (day 7; gray dashed line). Top: growth of *T. pseudonana* under axenic conditions and in cultures with each bacterial strain. Bottom: growth of bacterial strains in co-culture with *T. pseudonana*. *n* = 3; error bars represent ±1 standard deviation.

Identification of ecologically relevant exometabolites was carried out in co-culture systems in which marine phytoplankter *T. pseudonana* CCMP1335 served as the sole carbon source for three bacterial strains. *R. pomeroyi* DSS-3, *Stenotrophomonas* sp. SKA14, and *P. dokdonensis* MED152 were individually inoculated into a *T. pseudonana* culture that had accumulated exometabolites over 7 days. Bacterial gene expression in response to compounds in the exometabolite pool was identified after 8 h by comparison to gene expression in a single-substrate (glucose) control. Drawdown of *T. pseudonana* exometabolites in the co-culture spent medium was analyzed after 8, 24, and 48 h by LC-MS (Table S4) [41] and after 48 h incubations by 2D HSQC NMR (Holderman et al. in prep.). The control for the chemical analyses was spent medium from axenic *T. pseudonana* cultures. Microscopic observations did not reveal close physical attachment of the bacteria to diatom cells, although *P. dokdonensis* appeared associated with diatom extracellular polysaccharides after 48 h in co-culture. There was no evidence of altered growth rates by the diatom in the presence of the bacteria (Kruskal Wallis, *p* = 0.30, *n* = 3, *T* = 253 h; Fig. 1).

### *Ruegeria pomeroyi* DSS-3 metabolite utilization

**Fig. 2:**
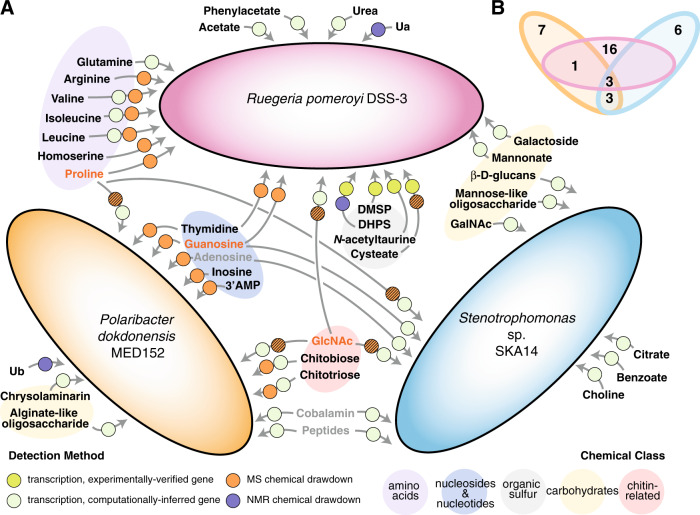
Metabolites involved in autotroph-heterotroph carbon transfer for three model marine bacteria when co-cultured with *T. pseudonana*. **A.** Summary of results from exometabolite chemical analysis and bacterial gene expression. Black font indicates metabolites unique to a single strain; gray font indicates metabolites shared by two strains; and orange font indicates metabolites shared by three strains. Circles specify the detection method(s), with hatched symbols indicating a mismatch between expression and drawdown methods. Metabolites Ua and Ub are unidentified compounds detected by ^13^C-HSQC NMR. **B** Venn diagram summary of unique and shared metabolites.

**Fig. 3:**
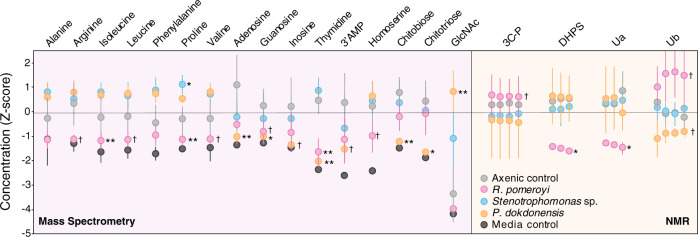
Bacterial drawdown of diatom exometabolites compared to axenic diatom cultures based on analysis of spent media by targeted LC-ESI-MS (left) and ^13^C-HSQC NMR (right). Data are *Z* scores of adjusted concentration (MS analysis) and scaled area (NMR analysis). Grouped data points indicate linked carbon atoms in NMR analysis. See Fig. S1 for time course of MS data and Fig. S2 for contour plots of NMR features. *n* = 3–7. ** = treatments significantly different from the axenic diatom cultures at adjusted *p* ≤ 0.01; * = treatments different from the axenic diatom cultures at *p* ≤ 0.05; † = treatments different from the axenic diatom cultures at *p* < 0.10.

*R. pomeroyi* transporters that were enriched in the co-cultures compared to the glucose control included ABC and TRAP transporters, both employing dedicated solute binding proteins that recognize substrates with high affinity, as well as permeases that depend largely on diffusion. Five enriched transporters had hypothesized annotations for amino acid uptake (Fig. 2 and Table S5). Spent medium drawdown patterns were consistent with this, and specifically indicated that concentrations of arginine and the branched chain amino acids valine, leucine, and isoleucine were lower in co-cultures with *R. pomeroyi* compared to axenic *T. pseudonana* cultures (Fig. 3). Nucleoside uptake was also indicated by spent medium drawdown, with significantly lower concentrations of guanosine and thymidine (Figs. 3 and S1).

Organic sulfur substrates indicated by transcript enrichment included 2,3,-dihydroxypropane-1-sulfonate (DHPS) and cysteate and *N*-acetyltaurine each containing a nitrogen atom as well (Tables 1 and S5). For all of these, the transporter genes have been verified experimentally in *R. pomeroyi* (Table 1). In the case of DHPS, spent medium drawdown analysis further indicated bacterial consumption based on significantly lower concentrations in the co-cultures. Drawdown analysis also indicated homoserine consumption (Fig. 3). Although the transporter for dimethylsulfoniopropionate (DMSP) has not yet been identified in the *R. pomeroyi* genome, enrichment of transcripts for functionally verified genes *dmdA* (DMSP demethylase) [47] and *acuI* (acrylyl-CoA reductase) [48] (Tables 1 and S5) suggested uptake of this organic sulfur metabolite.

**Table 1:** Metabolites hypothesized to support bacterial heterotrophy in diatom co-cultures based on significant changes in bacterial gene expression (adjusted *p* < 0.05) or metabolite concentrations in spent media (adjusted *p* < 0.10) for three bacterial strains, and the methodological approach providing support.

Substrate	*R. pomeroyi*	*Stenotrophomonas*	*P. dokdonensis*	References
Organic nitrogen				
3′ AMP			M	
Adenosine		G	M	
Arginine	M			
Chitobiose			M, G	
Chitotriose			M, G	
Choline		G		
* N*-acetyl-d-galactosamine		G^a^		[53]
* N*-acetyl-d-glucosamine	G	G	G	
Glutamine	G			
Guanosine	M	G	M	
Homoserine	M			
Inosine			M	
Isoleucine	M, G			
Leucine	M, G			
Peptides		G	G	
Proline	M	G	G	
Thymidine	M		M	
Urea	G			
Valine	M, G			
Organic nitrogen and sulfur				
Cysteate	G^a^			[98]
* N*-acetyltaurine	G^a^			[99]
Organic sulfur				
DHPS	M, G^a^			[100]
DMSP	G^a^			[47, 48]
Carbon only				
Acetate	G			
Alginate-like oligosaccharide			G	
β-D-Glucans		G		
Benzoate		G		
Chrysolaminarin			G	
Citrate		G		
Mannonate	G			
Mannose-like oligosaccharide		G		
Phenylacetate	G			
Galactoside	G			
Others				
Cobalamin		G	G	
Ua	M			
Ub			M	

Metabolites Ua and Ub are unidentified compounds detected by ^13^C-HSQC NMR (Fig. S2).

*M* uptake suggested from metabolomics analysis, *G* uptake suggested from gene expression.

^a^Gene function is experimentally verified.

For five enriched transporter systems, computationally inferred target substrates included urea, phenylacetate, mannonate, acetate, and a galactoside. Transcriptome analysis indicated uptake of six additional metabolites by transporters with general (e.g., sugar and dicarboxylate) or no substrate annotation (Fig. 2 and Table S5). Drawdown analysis revealed a lower concentration of one unidentified metabolite (designated Ua). In two cases there were higher concentrations of compounds in the co-culture spent media (phosphate-containing three carbon compound 3C-P and Ub; Fig. 3), suggesting either release of these unidentified metabolites by *R. pomeroyi* or enhanced export by *T. pseudonana* in the presence of the bacterium. Overall, the transcriptome and spent medium analyses of the *T. pseudonana*–*R. pomeroyi* co-culture indicated that at least 20 components of the diatom exometabolite pool were used by this bacterium, 13 of which were organic nitrogen molecules and 4 of which were organic sulfur molecules.

### *Stenotrophomonas* sp. SKA14 metabolite utilization

Of the 23 organic compound transporters significantly enriched in the *Stenotrophomonas* sp. SKA14 transcriptome, six are TonB-dependent transporters (TBDTs) functioning in the outer membrane to bring molecules into the periplasm using energy from TonB proteins located in the inner membrane (Table S6); subsequent passage of substrates into the cytoplasm is typically carried out by permeases or other types of inner membrane transporters. Among the enriched TBDTs, three are annotated as cobalamin/B_12_ receptors and likely involved in vitamin acquisition. Other types of enriched transporters indicate uptake of peptides (major facilitator superfamily (MFS) and oligopeptide family transporters), amino acids (two permeases, a serine/alanine/glycine transporter), and benzoate (Fig. 2 and Table S6). However, there were no decreases in amino acids in the spent medium compared to the *T. pseudonana* axenic control. An actual increase in proline and phenylalanine concentrations (Fig. 3) suggested either release of these metabolites by *Stenotrophomonas* or their enhanced export by *T. pseudonana* when *Stenotrophomonas* was present. Transporters for purines (MFS transporter) and nucleosides (NupC) were upregulated in co-culture (Table 1).

**Fig. 4:**
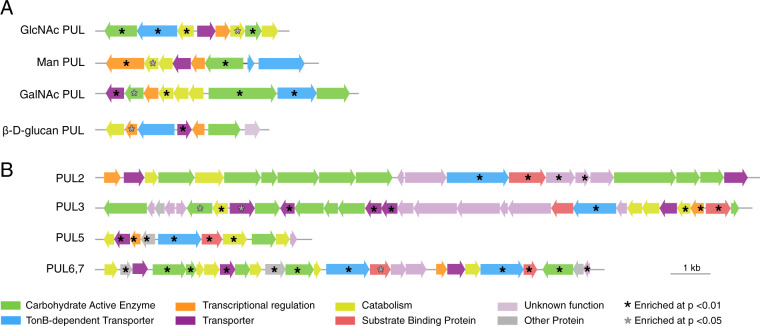
Polysaccharide utilization loci (PULs) containing genes enriched in diatom co-cultures relative to glucose controls. **A** *Stenotrophomonas* sp. SKA14. **B** *Polaribacter dokdonensis* MED152. Gene annotations and fold-change data are given in Tables S6 and S7.

Carbohydrate utilization was important in the *Stenotrophomonas* co-cultures and accomplished largely via polysaccharide utilization loci (PULs), genomic regions that provide Gammaproteobacteria [7] and Flavobacteriales [49] with the capacity to hydrolyze polysaccharide structures while retaining the resulting monomers in the periplasm until passage inside the cell. Both TBDTs and carbohydrate degrading enzymes (CAZymes) are common in these regions. Among the four PULs containing enriched genes in the *Stenotrophomonas* co-culture transcriptome is a likely *N*-acetyl-d-glucosamine (GlcNAc) PUL (Fig. 4) containing an enriched glycoside hydrolase (GH) that acts on *N*-acetylglucosides (GH-20), a glucokinase that phosphorylates glucose, a sugar membrane permease, and several GlcNAc catabolic enzymes (such as *nagA*) [50, 51]. The two chitinases also present in this PUL were not enriched, a pattern suggesting that *Stenotrophomonas* sp. SKA14 was targeting GlcNAc or chitin oligosaccharides in the *T. pseudonana* exometabolite pool. The hypothesized mannose oligosaccharide PUL system includes two GHs annotated as a β-mannosidase (GH2) and α-1,2-mannosidase (GH92). The likely *N*-acetyl-d-galactose PUL (GalNAc PUL) has a gene content consistent with activity toward galactose-containing carbohydrates [52–54], and is homologous to an operon in *Stenotrophomonas maltophilia* K279a verified experimentally to catabolize *N*-acetyl-d-galactosamine [53]. Significantly enriched genes in the co-culture transcriptome (Fig. 4) included an MFS transporter that brings *N*-acetyl-d-galactosamine through the inner membrane (AgaP), a tagatose-biphosphate aldolase (AgaY) that carries out the final step in *N*-acetyl-d-galactosamine catabolism, and a GH containing a carbohydrate binding module with activity against substrates such as galactose oligosaccharides, galactomannans, and galactolipids (Table S6). Finally, a likely 1,3/1,4-β-d-glucan PUL contains an upregulated exo 1,3/1,4-β-d-glucan glucohydrolase (GH3) predicted to hydrolyze residues of β-d-glucans [55].

There were also seven carbohydrate-active enzymes located outside PUL regions among the enriched *Stenotrophomonas* sp. genes (Table S6), two of which have computationally derived annotations for utilization of choline and chitin. Overall, transcriptomes and drawdown assays hypothesized at least 12 diatom metabolites serving as substrates for *Stenotrophomonas* in the co-cultures (Table 1), 7 of which contain nitrogen and 4 of which are carbohydrates.

### *Polaribacter dokdonensis* MED152 metabolite utilization

In the co-culture transcriptomes of *P. dokdonensis*, components of 13 organic molecule transporters were significantly enriched compared to the glucose control (DeSeq2; *p* < 0.01) (Fig. 2 and Table S7). Six of these are TBDTs, most located in PULs with carbohydrate utilization functions but one annotated for cobalamin uptake. Of the seven other transporters, one is a general MFS transporter with no substrate information, and one is annotated as a putative amino acid transporter. Exometabolite analysis showed no evidence of amino acid drawdown in the *P. dokdonensis* co-culture, but did indicate decreases in concentrations of inosine, adenosine, thymidine, and guanosine (Fig. 3). Peaks putatively assigned to unidentified metabolite Ub were also drawn down in the *P. dokdonensis* co-culture relative to axenic *T. pseudonana*.

Carbohydrate utilization via PULs was also important in the *P. dokdonensis* co-cultures. PUL2 (designations from the CAZy database) [55] (Fig. 4) is predicted to degrade chrysolaminarin, a glucose storage glucan synthesized by diatoms [56, 57] that is ubiquitous in ocean organic matter pools [58]. The enriched substrate binding proteins (*susD*) is an ortholog of a Flavobacterales gene demonstrated to bind laminarin [59], a storage glucan similar to chrysolaminarin found in brown macroalgae. For PUL3 of *P. dokdonensis* (Fig. 4), we hypothesize a function in transport and catabolism of fucose-like or maltooligosaccharide-containing compounds based on inner membrane transporters (Table S7). PUL5 was the most enriched PUL in the *P. dokdonensis* transcriptome, and we predict it transports and catabolizes GlcNAc in the form of chitin monomers or oligomers. This PUL aligns modularly with a Bacteroidaceae PUL in a bacterium confirmed to grow on GlcNAc [60], and the enriched MFS transporter (Table S7) is an ortholog of a gene in the GlcNAc PUL of a *Stenotrophomonas* species. Genes encoding chitin hydrolysis were not found in the *P. dokdonensis* genome, suggesting this bacterium is only able to use monomers and oligomers of chitin. Bacterial drawdown analysis agreed with this annotation by indicating significantly lower concentrations of chitobiose and chitotriose in the co-culture spent medium (Fig. 3), but concentrations of GlcNAc were higher. PUL6 and PUL7 are co-located (Fig. 4) and we predict they target glycans containing guluronic acid and mannuronic acid, the two primary monomers of alginate. While alginate has not been reported as a component of *T. pseudonana* polysaccharides, guluronic acid is present in diatom cell walls [61] and the *P. dokdonensis* PUL6 is highly syntenic with those of previously described alginate-catabolizing Flavobacteriales [62–64]. The enriched components of PUL6 and PUL7 include a poly(β-d-mannuronate) lyase and two alginate lyases, also supporting a function for this genomic region in utilization of an alginate-like polysaccharide (Table S7). Nine enriched CAZymes that were not associated with PULs included a gene with catabolic activity against β-1,3 glucans, a gene with activity toward various β-1,3 and β-1,4 glucans, and an alginate lyase. A total of 14 possible substrates were identified for *P. dokdonensis*, 10 of which were organic nitrogen compounds and 5 of which were carbohydrates (Table 1).

## Discussion

Metabolites passed between marine microbes play key roles in ocean ecosystems, influencing bacterial community assembly and diversity [65, 66], mediating competition, antagonism, and mutualisms [25], and serving as currencies of carbon flux. The last of these roles is a central function in global carbon cycling by which ~50% of marine photosynthate is transferred from phytoplankton to heterotrophic bacteria via the labile DOC pool [1, 67, 68]. There are two primary reasons why little is known about the metabolites linking marine phytoplankton and bacteria [69]. One is that the most biologically reactive components of marine DOC do not accumulate and therefore account for an extremely small fraction of the total reservoir [1]; and the other is that direct chemical analysis, especially in a seawater matrix, is challenging unless the identity of a compound is already known [70].

Our approach delineated clear differences in resource use patterns by bacteria individually inoculated into identical exometabolite pools formed during exponential through stationary growth phases of a diatom. Of the 36 molecules (Fig. 2) covering chemical classes ranging from amino acids to organic sulfur compounds, monosaccharides, oligosaccharides, nucleosides, and organic acids, 29 (80%) were targeted for uptake up by only one of the three representative bacterial strains. Rhodobacterales member *R. pomeroyi* was hypothesized to uniquely utilize four amino acids, the organic sulfur compounds DHPS, cysteate, DMSP, and *N*-acetyltaurine, the carboxylic acids acetate and phenylacetate, two carbohydrates, and urea, suggesting a substrate suite that is diverse, dominated by LMW compounds, and distinguished by a focus on organic sulfur metabolites. Gammaprotebacteria member *Stenotrophomonas* sp. SKA14 was hypothesized to uniquely utilize three carbohydrates plus benzoate, citrate, and choline. Flavobacteriales member *P. dokdonensis* uniquely utilized the chitin oligomers chitobiose and chitotriose, the carbohydrate storage compound chrysolaminarin, an alginate-like carbohydrate, 3′AMP, and the nucleoside inosine. The latter two bacteria had substrate use linked to polymeric and oligomeric carbohydrates that, particularly in the case of *P. dokdonensis*, is consistent with significant roles in aging blooms [11, 23]. Low overlap in the utilization of labile organic matter has been proposed to explain the predictable co-occurrence of Rhodobacterales, Gammaproteobacteria, and Flavobacteriales with microphytoplankton in the surface ocean [11, 23, 27, 71, 72]. Our analyses concur that substrate overlap is low among these groups when processing a natural pool of phytoplankton-derived molecules. Furthermore, they point to resource-based niche partitioning of the available resources, rather than competition for them, as the underlying ecological explanation.

Seven of the 36 metabolites, however, were targeted for uptake by more than one bacterial strain: proline, GlcNAc, peptides, cobalamin, and the nucleosides adenosine, guanosine, and thymidine. All of these are organic nitrogen compounds and one is also a vitamin, suggesting these may be resources for which there is competition among the marine bacteria growing at the expense of diatom exometabolites. Proline, guanosine, and GlcNAc were targeted for uptake by all three strains, with the availability of GlcNAc possibly linked to the chitin present in diatom cell walls [73]. Organic nitrogen compounds were also common in the non-shared metabolites, making up ~45% of metabolites.

Previous studies of endometabolite composition of diatom cells reported some of the same compounds identified here in the *T. pseudonana* exometabolome (Table 1). These include the amino acids glutamine, arginine, valine, isoleucine, leucine, and proline [74, 75], the organic sulfur compounds DMSP and DHPS [76, 77], choline [74, 78], galactose [79], and chrysolaminarin [77]. Previous studies have also shown chemotaxis by marine bacteria to several compounds identified in the *T. pseudonana* exometabolome. These include valine, proline, DMSP, and GlcNAc [80, 81]. Evidence that these compounds are present in diatom cells, function as attractants in chemotaxis, and are taken up differentially by heterotrophic bacteria suggests they play roles in the chemical ecology of bacterial community assembly. Niche dimensions unrelated to carbon and nutrient acquisition were not examined here, but may also influence ecological differentiation among co-existing bacterial species in the surface ocean; such factors include physical conditions [82], growth kinetics [83], antimicrobial defenses [84], and death processes through viral and protist grazing [85].

Some metabolites had different patterns in gene expression compared to drawdown. Examples are the enrichment of transcripts from the experimentally verified cysteate transporter in *R. pomeroyi* (Tables 1 and S5) but no evidence of cysteate release by axenic diatoms; and enrichment of transcripts from possible proline transporters in both *Stenotrophomonas* sp. and *P. dokdonensis* (Tables 1, S6, and S7) but no evidence of proline drawdown for either (Fig. 3). Differential exometabolite release by the diatom in axenic versus co-culture (i.e., different composition or rate of exometabolite release in the presence versus absence of bacteria) could explain these methodological mismatches. There is growing evidence that marine phytoplankton can detect the presence of bacteria, including a potential recognition cascade invoked by *T. pseudonana* when growing with *R. pomeroyi* [86]. Other scenarios that could lead to mismatches between these methodological approaches include lower bacterial drawdown rates than diatom release rates, and bacterial metabolite release. A potential example of the last scenario is the accumulation of GlcNAc in the *P. dokdonensis* co-culture but not the axenic diatom culture. In this case, *Polaribacter* hydrolysis of chitobiose and chitotriose (Fig. 3) may be outpacing its uptake of the hydrolysis product GlcNAc [87].

The interpretation of gene expression patterns used here assumed that upregulation of transport and catabolic genes was stimulated by the availability of a substrate. This is based on the unfavorable energetics of across-the-board synthesis of transporter systems that may not yield benefits [88], as well as previous observations of transporter expression changes when the substrates available to heterotrophic marine bacteria are manipulated [26, 89, 90]. Other regulatory strategies are possible, however, such as constitutive regulation, posttranscriptional regulation, or co-regulation, and these may not be detected in this study or could mislead interpretation. Finally, gene expression interpretations assume that computationally inferred annotations of transporter and catabolic genes in the bacterial genomes are generally correct, which is not always the case [91]. Across the three bacterial genomes, half the upregulated transporters have either general annotations or no annotations regarding their target substrates (Tables S5, S6, and S7), emphasizing the limitation of comparative genome analysis alone to address bacterial resource dimensions.

The role of resource competition in determining bacterial community assembly has been explored recently both in experimental systems and metabolic models [66, 92–94]. Yet current knowledge of microbial metabolites is hindered by barriers to capturing and identifying microbial products, and further exacerbated by the difficulty of microbial gene annotation. Thus it is challenging to go beyond generic products of central metabolism in addressing resource-based bacterial niche dimensions. Indeed, metabolites of noncore processes that are by and large missing from experiments and models may be the compounds most likely to support resource-based niche differentiation. For example, DHPS release by marine diatoms (Fig. 2) was mentioned only two times in the oceanographic literature [95, 96] before it emerged as a major resource for certain taxa of heterotrophic bacteria [26, 97]. Two types of information are critical for improving understanding of bacterial resource partitioning. The first is a chemical database that captures the diversity of microbial metabolites. The second is experimental annotation of bacterial transporter genes that hold important clues about resource-based niche dimensions in the surface ocean [69].

High turnover rates and low concentrations make identification of the labile organic matter released by phytoplankton problematic. Here we used biological vetting based on bacterial activity to identify the molecules most likely to shape heterotrophic bacterial communities reliant on recent photosynthate. Progress in closing the knowledge gap of marine metabolites will enable new insights into the transfer of carbon between major ocean reservoirs.

## Supplementary information

Supplemental Material

Supplemental Material Table S1

Supplemental Material Table S2

Supplemental Material Table S3
