# Supplementary material for: Resource partitioning of phytoplankton metabolites that support bacterial heterotrophy
Source: ISME J. 2020 Oct 23;15(3):762–73. doi: 10.1038/s41396-020-00811-y (PMC8027193; doi:10.1038/s41396-020-00811-y)
Supplement: Supplementary file 1 — Supplemental Material [file 41396_2020_811_MOESM1_ESM.pdf]

Resource Partitioning of Diatom Metabolites that Support Bacterial Heterotrophy in the Ocean

Frank Xavier Ferrer-González, Brittany Widner, Nicole R. Holderman, John Glushka, Arthur S. Edison, Elizabeth B. Kujawinski, and Mary Ann Moran

Supplementary Methods:

Three bacterial strains were introduced individually into 7 d diatom co-cultures. Samples were collected after 8 h, 24 h, and/or 48 h and analyzed for bacterial response via transcriptomics (to measure regulation changes) or via MS or NMR (to measure metabolite drawdown) (Fig. 1).

Co-cultures

Axenic cultures of *Thalassiosira pseudonana* 1335 culture (National Center for Marine Algae) were grown in organic-carbon free medium L1 +Si (1) in 1900 ml vented polystyrene tissue culture flasks at 18°C under 16 h light at 160 µmol photons $m^{-2}s^{-1}$ and 8 h dark. For samples used in NMR analysis, ^{13}C bicarbonate was used to make the L1 medium. After the diatom cultures had been growing for 7 d, bacteria pre-grown in YTSS medium were washed 5 times in sterile L1 medium and inoculated into the diatom cultures at ~10^{6} (*Stenotrophomonas* sp. and *P. dokdonensis*) or ~10^{7} (*R. pomeroyi*) cells ml^{-1}. Three replicate co-cultures were established for each bacterial strain. After incubation in the light for 8 h, *T. pseudonana* cells were removed by pre-filtration through polycarbonate 2.0 µm pore-size filters, and bacteria were collected on 0.2 µm pore-size Supor filters. Filters were immediately flash frozen in liquid nitrogen and stored at -80°C until processing, and the filtrate was stored frozen for subsequent chemical analysis. Filtrate was also obtained 24 h and 48 h after bacterial inoculation for chemical analysis. An additional treatment was established in which strains were introduced individually into a defined medium with glucose as the carbon source (L1 medium +Si with 2.5 mM glucose), and this served as the control treatment for co-culture transcriptome analysis (Fig. 1). Bacterial strains were similarly inoculated into the medium, collected on 0.2 pore-size filters after 8 h, and flash frozen.

Cell counts

Culture samples were fixed to a final concentration of 1% glutaraldehyde, incubated at 4°C for 20 min, and stored at −80°C. Just prior to analysis, an internal standard of 5 µm fluorescent beads was added (Spherotech, Lake Forest, IL, USA), followed by stained for 15 min with SYBR Green I (final

concentration 0.75X; Life Technologies, Waltham., MA, USA). Samples were analyzed on an Agilent Quanteon flow cytometer (Acea, Biosciences Inc, San Diego CA). Flouorescence was detected with a 405 nm laser using a 530/30 bandpass filter for SYBR Green (bacteria) a 695/40 bandpass filter for chlorophyll a. There was no bacterial contamination of axenic cultures based on scattergrams from flow cytometry and aliquots from axenic cultures spread onto YTSS plates.

RNA-seq Analysis

Filters were cut into pieces using sterile technique and incubated at room temperature for 1 h in TE buffer, SDS (0.6% final concentration), and proteinase K (120 ng µl $^{-1}$ final concentration). An equal volume of acid phenol:chloroform:isoamyl-alcohol was added and samples were shaken, incubated for 15 min, and centrifuged at 4°C for 15 min for collection of the supernatant. This process was repeated on the supernatant with addition of an equal volume of chloroform:isoamyl-alcohol. The resulting supernatant was mixed with 1 volume of isopropanol and sheared by passage through a 21 g syringe needle. Samples were incubated overnight at -20°C, centrifuged at 4°C for 30 min, washed twice with 75% ethanol, and dried and resuspended in RNAse-free water. Potential traces of DNA were removed using the Turbo DNA-free kit (Invitrogen, Waltham, MA, USA). Samples were tested for residual DNA by a 40-cycle PCR targeting the 16S rRNA gene of the bacterial strains. Duplicates of each treatment were randomly selected for carrying forward through rRNA depletion using the Ribo-Zero Bacteria kit (Illumina, San Diego, CA, USA), library preparation using the KAPA Stranded RNA-Seq Kit (Kapa Biosystems, Wilmington, MA, USA), and sequencing on a HiSeq Illumina 2500 at the Hudson Alpha Institute for Biotechnology (Huntsville, AL, USA). Raw data were deposited in the NCBI SRA BioProject database under accession PRJNA448168.

Quality control was performed on 207 million 50-bp reads using the FASTX toolkit, imposing a minimum quality score of 20 over 80% of read length. Reads aligning to an in-house rRNA database were removed (blastn, score cutoff $\geq$ 50). Remaining reads were mapped to the *R. pomeroyi*, *Stenotrophomonas* sp. SKA14, or *P. dokdonensis* MED152 genomes (Bowtie 2; (2) and counted (HTSeq; (3), conserving strand information and removing reads that mapped to more than one location. Genes with differential expression were determined with DESeq2 (4). Annotations of the bacterial genomes were updated by comparison to closely-related genomes in the IMG (5) and NCBI (6, 7) databases, and to a database of TnSeq derived annotations (8). The dbCAN web resource was used for identification of carbohydrate-active enzyme annotations, taking into account results of HMMs, peptide pattern

recognitions, and protein alignments (9). Reciprocal best hit analysis was carried out against genomes of well-annotated Gammaproteobacteria (*Stenotrophomonas maltophila* K279a), and Flavobacteriia (*Formosa sp.* Hel_1_33_133, *Bacteriodetes ovatus* ATCC8433, *Gramella forsetii* KT0803, and *Zobellia galactanivorans* DsijT).

Mass Spectrometry and NMR Analysis

Chemical analysis was conducted on filtered spent media from the co-cultures and axenic *T. pseudonana* culture and on uninoculated L1 as the medium blank. For mass spectrometry analysis, 8, 24, and 48 h co-culture spent media were analyzed. Metabolites were derivatized with benzoyl chloride (Widner et al. in prep) by modification of methods from Oehlke et al. (10) and Wong et al. (11), extracted using a solid phase resin (Agilent, Bond Elut PPL), and analyzed using ultra high performance liquid chromatography coupled with electrospray ionization and tandem mass spectrometry (UHPLC-ESI-MSMS) with modifications to Kido Soule et al. (12). Unless otherwise noted, all samples and reagents for mass spectrometry analysis were stored and transferred with acid-washed (10% HCl), combusted (450°C for 5 hours) glassware, and all solvents were Thermo Fisher Optima Grade. Standards were obtained from Sigma-Aldrich at the highest purity available. Filtered (0.2 µm) samples were divided into two 1 mL aliquots and one was spiked with a standard mix (100 ng/mL final concentration; Table S4) to correct for matrix effects. Each sample was combined with 300 µL sodium carbonate, 30 µL sodium hydroxide, and 200 µL working reagent (5% benzoyl chloride in acetone V:V). After vortexing for 5 min, samples were acidified with concentrated phosphoric acid to pH 2-3 (15 µL). The acetone was evaporated using a vacufuge (Eppendorf), and an equivalent volume of Milli-Q water was added to replace the acetone. One mL of the aqueous phase was loaded onto solid phase extraction cartridges (1g/6ml, Bond Elut PPL, Agilent, conditioned according to manufacturer's specifications). Samples were eluted from the cartridge with methanol (6 mL), dried down (vacufuge), reconstituted in 90:10 water:acetonitrile (100 µL), and stored at -20 ^{o}C until analysis.

Mass spectrometry samples were analyzed on a Vanquish UHPLC system [Waters Acquity HSS T3 column (2.1 x 100 mm, 1.8 µm) with a Vanguard pre-column at 40 ^{o}C] coupled via heated electrospray ionization (H-ESI) to an ultrahigh resolution tribrid mass spectrometer, the Orbitrap Fusion Lumos (Thermo Fisher Scientific). The column was eluted with mobile phase (A) 0.1% formic acid in water and (B) 0.1% formic acid in acetonitrile at a flow rate of 0.5 mL min^{-1}. The gradient conditions were 0-0.5 min (1% B), 2 min (10% B), 2-5 min (10% B), 7 min (25% B), 7-9 min (25% B), 12.5 min (50% B), 13 min (95%

B), 13-14.5 min (95% B). The system was then returned to 1% B for re-equilibration prior to the next injection (total gradient time 16 min). Separate injections of 5 µL each were made for positive and negative ion modes. The electrospray voltages were 3600 V (positive) and 2600 V (negative), and the source gases were 55 (sheath) and 20 (auxiliary), and 1 (sweep). The capillary temperature was 350 ^{o}C and the vaporizer temperature was 400 ^{o}C. The Lumos was operated in full-scan MS mode with data-dependent tandem mass spectrometry, guided by a list of user-defined parent ions with a retention time window for each (full MS/ddMS2 with inclusion list). In this targeted quantitation approach, data are collected in full-scan mode in the Orbitrap analyzer (resolution 60,000 fwhm, at m/z 200). When any of the parent ions on the inclusion list are detected within the retention time window, MS/MS scans are automatically acquired (resolution 7,500 fwhm, at m/z) and then the instrument resumes full-scan operation. Data obtained in full-scan mode were used for detection and quantification, while MS/MS data were used for identification and confirmation. Full-scan MS data were collected between 170-1000 m/z, and the automatic gain control (AGC) setting was 4e5 with a max injection time of 50 msec. MS/MS data were collected using higher energy collisional dissociation (HCD) with collision energy at 35% and intensity threshold at 2e4. The MS/MS AGC target setting was 5e4 with a max injection time of 22 msec. Parent ions were isolated within the quadrupole at an isolation width of 1 m/z. All data were collected in profile mode. See Table S4 for retention times and parent and product ion values.

Standards were prepared in duplicate in L1 medium from 0.5 to 500 ng/mL. Sample and standard peaks were integrated using Skyline (13, 14), and standard curves were calculated using at least 5 standards. Standard curve R squared values were required to be greater than 0.92 for metabolite quantification (Table S4). In preliminary experiments (Widner et al. in prep), we determined that the instrument response could be enhanced or suppressed in some spent culture media relative to the standards in L1 medium. The analytical recoveries were calculated as the difference between the calculated concentrations of the spiked sample (sample concentration + 100 ng/mL spike) and the un-spiked sample divided by the spike concentration (100 ng/mL). The concentration of each sample was corrected for matrix effects by multiplying the raw concentration by the average analytical recovery for that culture media type. Because of these corrections, we are reporting ‘adjusted concentration’ for metabolites.

Mass spectrometry metabolites were evaluated statistically in Matlab by comparing adjusted sample concentrations (from all time points) by treatment using a one-way ANOVA ($\alpha = 0.05$) and post-

hoc Dunnett's test to compare each co-culture to the axenic *T. pseudonana*. Outliers were defined as values that exceeded 3 scaled median absolute deviations and were excluded from statistical analysis.

For NMR analysis, 5 ml of 48 h co-culture spent medium were lyophilized, homogenized dry (MP Biomedicals FastPrep-96) through 3 x 30 s cycles at 1800 rpm using 5 x 3.5 mm glass beads, reconstituted in 200 μL dimethyl-sulfoxide-d6 (DMSO-d6) with 50 μM sodium trimethylsilylpropanesulfonate-d6 (DSS-d6, Cambridge Isotope Laboratories, Inc.) and re-homogenized through 3 x 30 s cycles at 1800 rpm. Following centrifugation for 10 min at 22 ^{o}C and 5000 x g, the supernatant was centrifuged for 15 min at 5000 x g and added in 40 μL aliquots to 1.7 mm NMR tubes in a 96 tube rack. Two-dimensional HSQC-NMR spectra were collected (Bruker 800 MHz NEO with 1.7 mm cryoprobe) with an automatic and refrigerated (279 K) sample changer (SampleJet) using IconNMR (V5.1). Before NMR data acquisition, samples were preincubated for 5 min in the probe for temperature equilibration at 300 K. 2D data were collected using acquisition parameters modified from a hsqcetgpsisp2.2 pulse program (TopSpin V4.0.6). Spectra were acquired in Echo-Antiecho acquisition mode in 3 h 58 min with presaturation of residual water using 64 scans and 32 dummy scans. The indirect ^{13}C (f1) dimension had a spectral width of 90.0027 ppm, 128 data points, and an offset of 45 ppm. In the direct ^{1}H dimension (f2) had a spectral width of 13.0255 ppm, 4166 data points, and an offset of 3.691 ppm.

Spectra were processed in MNOVA by applying a 90° sine square apodization, 4K zero-filling, and Fourier-transformation. Transformed spectra were auto-phased, baseline-corrected and referenced along f1 and f2 to DSS-d6 (0.0, 0.0 ppm). All peaks above the noise were manually integrated. These peaks were divided into four sub-regions for visualization (Figure S2) and analysis using MATLAB. Raw data, peak lists, and analysis scripts are available on MetaboLights under accession MTBLS1544.

The peak integral values were imported into a MATLAB workflow to normalize, scale, and analyze spectral features. The peak integral table was normalized using built-in probabilistic quotient normalization (PQN), mean-centering, and univariate scaling functions. The built-in MATLAB principal components analysis (PCA) function was applied to feature integrals across co-culture conditions, and a loadings plot along principal component 1 was generated to determine which peaks accounted for the largest proportion of variance among samples. The loadings plot informed our decision for which peaks to analyze further, and all peaks between the covariance threshold of -0.5 and 0.5 were omitted from

further analysis as they accounted for the least between-sample variation. From these data, P-values, false discovery rates (FDR), and q-values were calculated using MATLAB built-in functions.

References

1. Guillard R, Hargraves P. *Stichochrysis immobilis* is a diatom, not a chrysophyte. Phycologia. 1993;32(3):234-6.
2. Langmead B, Salzberg SL. Fast gapped-read alignment with Bowtie 2. Nat Meth. 2012;9(4):357.
3. Anders S, Pyl PT, Huber W. HTSeq—a Python framework to work with high-throughput sequencing data. Bioinformatics. 2015;31(2):166-9.
4. Love MI, Huber W, Anders S. Moderated estimation of fold change and dispersion for RNA-seq data with DESeq2. Genome Biol. 2014;15(12):550.
5. Chen I-MA, Chu K, Palaniappan K, Pillay M, Ratner A, Huang J, et al. IMG/M v. 5.0: an integrated data management and comparative analysis system for microbial genomes and microbiomes. Nucleic Acids Res. 2018;47(D1):D666-D77.
6. O'Leary NA, Wright MW, Brister JR, Ciufo S, Haddad D, McVeigh R, et al. Reference sequence (RefSeq) database at NCBI: current status, taxonomic expansion, and functional annotation. Nucleic Acids Res. 2015;44(D1):D733-D45.
7. Tatusova T, DiCuccio M, Badretdin A, Chetvernin V, Ciufo S, Li W. Prokaryotic genome annotation pipeline. The NCBI Handbook [Internet] 2nd edition: National Center for Biotechnology Information (US); 2013.
8. Price MN, Wetmore KM, Waters RJ, Callaghan M, Ray J, Liu H, et al. Mutant phenotypes for thousands of bacterial genes of unknown function. Nature. 2018;557(7706):503.
9. Zhang H, Yohe T, Huang L, Entwistle S, Wu P, Yang Z, et al. dbCAN2: a meta server for automated carbohydrate-active enzyme annotation. Nucleic Acids Res. 2018;46(W1):W95-W101.
10. Oehlke J, Brudel M, Blasig IE. Benzoylation of sugars, polyols and amino acids in biological fluids for high-performance liquid chromatographic analysis. J Chromatogr B: Biomedical Sci Appl. 1994;655(1):105-11.
11. Wong J-MT, Malec PA, Mabrouk OS, Ro J, Dus M, Kennedy RT. Benzoyl chloride derivatization with liquid chromatography–mass spectrometry for targeted metabolomics of neurochemicals in biological samples. J Chromatogr A. 2016;1446:78-90.
12. Soule MCK, Longnecker K, Johnson WM, Kujawinski EB. Environmental metabolomics: Analytical strategies. Mar Chem. 2015;177:374-87.
13. Henderson CM, Shulman NJ, MacLean B, MacCoss MJ, Hoofnagle AN. Skyline performs as well as vendor software in the quantitative analysis of serum 25-hydroxy vitamin D and vitamin D binding globulin. Clinical Chem. 2018;64(2):408-10.
14. Pino LK, Searle BC, Bollinger JG, Nunn B, MacLean B, MacCoss MJ. The Skyline ecosystem: Informatics for quantitative mass spectrometry proteomics. Mass Spectrom Rev. 2017.

Supplementary Figures:

Figure S1. Time course of exometabolite concentrations based on targeted LC-ESI-MS analysis of axenic and co-culture spent media. ** = treatments significantly different from the axenic diatom cultures in the grouped analysis presented in Fig. 3 at $p \leq 0.01$; * = treatments significantly different from the axenic diatom cultures in the grouped analysis presented in Fig. 3 at $p \leq 0.05$ in Fig. 4. Error bars indicate standard deviations.

Figure S2. ^{13}C-HSQC NMR spectra of metabolite uptake in co-cultures. (A) Full representative spectra from *T. pseudonana* with insets B, C, D, and E. The structure of DHPS is shown with numbered peaks (D1, D2, and D3) corresponding to each C-H bond in the compound. (B-E) Detailed NMR signals of 18 features in axenic and co-culture spent media. Spectra from one of 3 replicate samples is shown. The peak labeled with an asterisk in panel C indicates a C – H bond in DHPS that lies within T1 noise.

Supplementary Tables:

Table S1. RNA-seq data for *R. pomeroyi* DSS-3. Log_2 fold-differences that are negative are enriched in the co-culture, differences that are positive are enriched in the glucose control.

Table S2. RNA-seq data for *Stenotrophomonas* sp. SKA14. Log_2 fold-differences that are negative are enriched in the co-culture, differences that are positive are enriched in the glucose control.

Table S3. RNA-seq data for *P. dokdonensis* MED152. Log_2 fold-differences that are negative are enriched in the co-culture, differences that are positive are enriched in the glucose control.

Table S4. Targeted metabolites that could be quantified by mass spectrometry in culture media with a calibration curve $R^2 \geq 0.92$. Each parent ion was fragmented into one or more product ions (Product 1, Product 2). N/A = absence of a product ion; # bz = number of benzoyl groups added to the target compound in the derivatization process; # Standards = number of standards in the calibration curve.

Table S5. Significantly enriched transporter systems and carbohydrate active enzymes in the transcriptome of *R. pomeroyi* DSS-3 in co-culture with *T. pseudonana*. ** $p < 0.01$, * = $p < 0.05$, NS = not

significant. GH = glycoside hydrolase, AA = auxiliary activity, CE = carbohydrate esterase. Alternating shading highlights multi-protein transporters.

Table S6. Significantly enriched transporter systems and carbohydrate active enzymes in the transcriptome of *Stenotrophomonas* sp. SKA14 in co-culture with *T. pseudonana* and their location in PULs. ** $p < 0.01$, * = $p < 0.05$. GH = glycoside hydrolase, CBM = carbohydrate binding module, AA = auxiliary activity, CE = carbohydrate esterase, GT = glycosyl transferase.

Table S7. Significantly enriched transporter systems and carbohydrate active enzymes in the transcriptome of *Polaribacter dokdonensis* MED152 in co-culture with *T. pseudonana* and their location in PULs. ** $p < 0.01$, * = $p < 0.05$. GH = glycoside hydrolase, CBM = carbohydrate binding module, AA = auxiliary activity, CE = carbohydrate esterase, GT = glycosyl transferase.

Figure S1

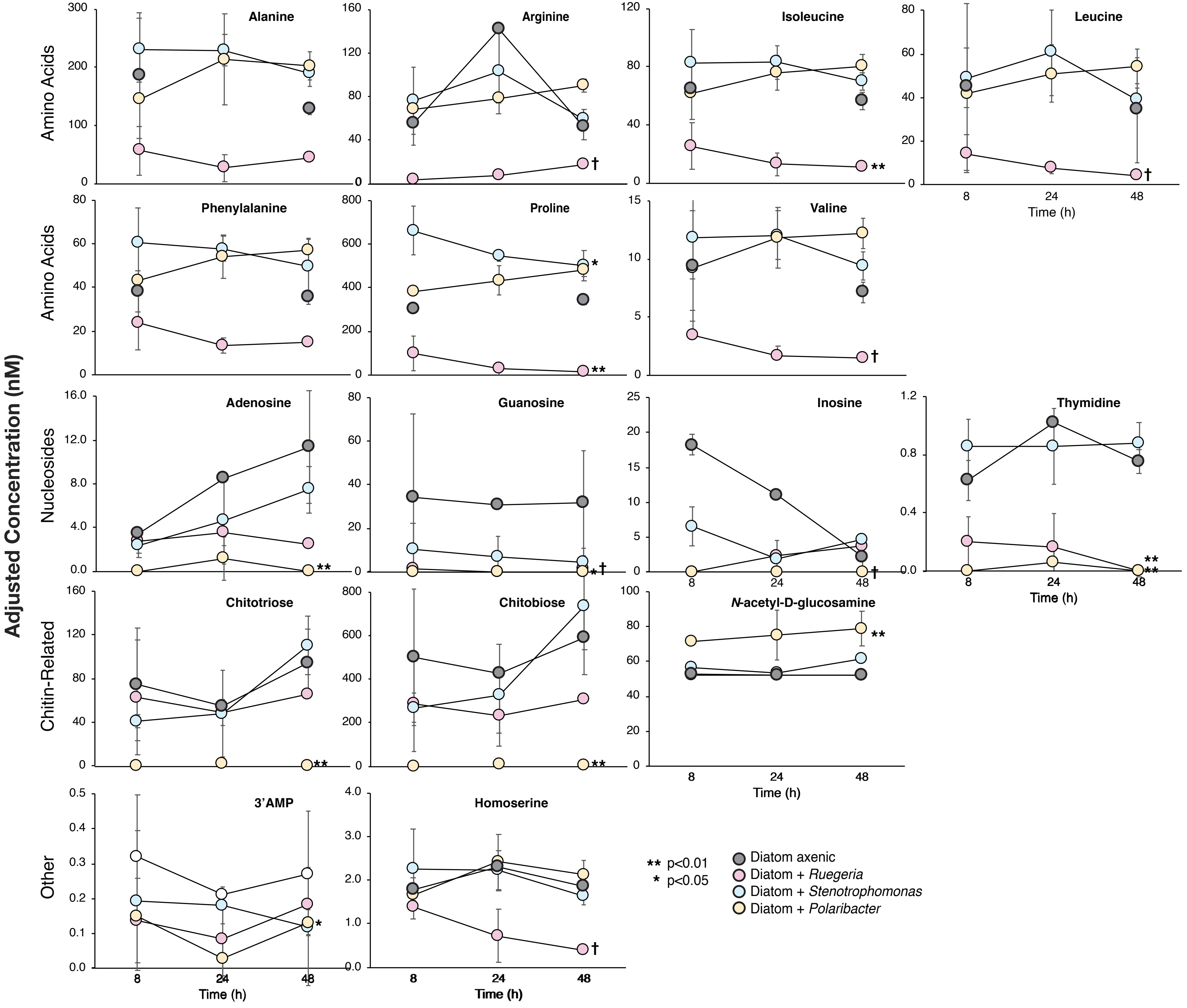
Adjusted Concentration (nM)
Amino Acids
Alanine
Arginine
Isoleucine
Leucine
Phenylalanine
Proline
Valine
Nucleosides
Adenosine
Guanosine
Inosine
Thymidine
Chitin-Related
Chitotriose
Chitobiose
N-acetyl-D-glucosamine
Other
3'AMP
Homoserine
Time (h)
8
24
48
** p<0.01
* p<0.05
Diatom axenic
Diatom + Ruegeria
Diatom + Stenotrophomonas
Diatom + Polaribacter

Figure S2

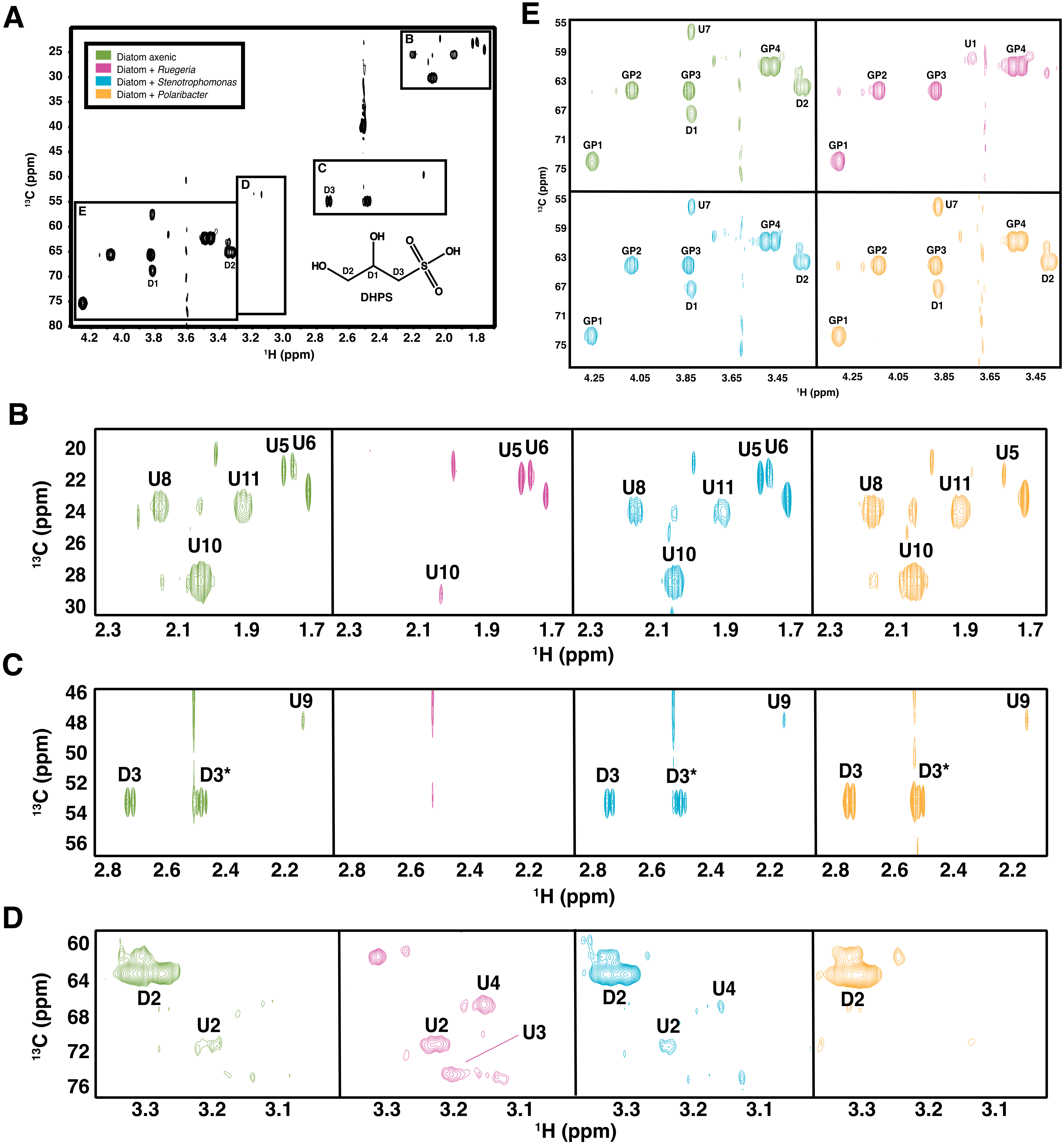

A
Diatom axenic
Diatom + *Ruegeria*
Diatom + *Stenotrophomonas*
Diatom + *Polaribacter*
B
C
D
E
D1
D2
D3
HO
OH
O
S
DHPS
¹³C (ppm)
¹H (ppm)
GP1
GP2
GP3
GP4
U1
U2
U3
U4
U5
U6
U7
U8
U9
U10
U11
D3*

Table S4. Targeted metabolites that could be quantified by mass spectrometry in culture media with a calibration curve R2 > 0.92. Each parent ion was fragmented into one or more product ions (Product 1, Product 2), where "N/A" indicates the absence of a product ion. "# bz" is the number of benzoyl groups added to the target compound in the derivatization process, and "Number of Standards" is the number of standards in the calibration curve.

Compound	Retention Time (min)	Parent (m/z)	Product 1 (m/z)	Product 2 (m/z)	Adduct	# bz	Correlation Coefficient (R2)	# Standards
2,3 -Dihydroxybenzoic acid	12.6	257.0455	121.0282	N/A	- H	1	0.950	8
2'deoxycitidine	7.8	332.1241	105.0330	216.0776	+ H	1	0.986	9
2'deoxyguanosine	8.5	370.1157	121.0281	150.0406	- H	1	0.983	7
3'AMP	5	450.0820	134.0456	78.9579	- H	1	0.987	7
4-aminobenzoic acid	10.9	240.0666	196.0762	N/A	- H	1	0.997	7
5'AMP	6.7	450.0820	134.0456	78.9579	- H	1	0.984	7
5'deoxyadenosine	7.7	354.1208	238.0727	N/A	- H	1	0.986	10
adenine	7.4	240.0880	105.0328	137.0449	+ H	1	0.978	7
adenosine	8.5	372.1302	105.0328	136.0612	+ H	1	0.938	7
alanine	6.6	192.0666	148.0762	120.0443	- H	1	0.996	8
AmMP	3.7	243.1240	122.0714	81.0445	+ H	1	0.988	5
arginine	3.3	279.1452	105.0330	220.0962	+ H	1	0.993	5
asparagine	3.4	237.0870	105.0328	122.0595	+ H	1	0.989	5
aspartate	4.5	236.0564	115.0023	120.0443	- H	1	0.996	8
chitobiose	7	529.2028	126.0543	204.0860	+ H	1	0.981	7
chitotriose	7	732.2822	126.0543	138.0550	+ H	1	0.986	7
ciliatine	3	230.0577	105.0328	95.0484	+ H	1	0.989	9
citrulline	4.6	278.1146	191.1194	235.1069	- H	1	0.992	8
cysteate	2.8	274.0380	166.9789	184.9895	+ H	1	0.964	6
cysteine	11.2	449.0846	105.0330	224.0368	+ H (dimer)	1	0.938	7
cytidine	7.4	346.1044	213.0661	135.0191	- H	1	0.996	5
desthiobiotin	7.3	215.1390	179.1161	197.1281	+ H	0	0.976	9
DHPS	5.4	259.0282	121.0285	N/A	- H	1	0.988	8
ectoine	5.4	265.1187	105.0330	98.0596	+ H	1	0.993	9
GABA	6.8	208.0968	105.0328	95.0484	+ H	1	0.993	5
glucosamine 6-phosphate	2.4	362.0646	78.9579	96.9681	- H	1	0.964	8
glutamic acid	5.5	250.0721	121.0290	162.0925	- H	1	0.997	6
glutamine	3.8	249.0881	120.0443	187.0880	- H	1	0.990	6
glycine	4.3	178.0510	132.0438	134.0607	- H	1	0.992	8
guanosine	8.2	388.1251	105.0328	152.0556	+ H	1	0.931	7
histidine	2.7	258.0884	120.0443	81.0452	- H	1	0.996	8
HMP	6.3	244.1080	105.0330	123.0456	+ H	1	0.940	7
homoserine	4.2	222.0772	120.0449	178.0872	- H	1	0.991	7
homoserine betaine	5	266.1387	105.0330	207.0643	+ H	1	0.995	5
inosine	1.9	269.0881	137.0449	N/A	+ H	0	0.939	7
isethionate	6.4	229.0176	121.0289	N/A	- H	1	0.977	6
isoleucine	11.4	234.1136	190.1247	82.0660	- H	1	0.988	8
kynurenine	11.2	313.1183	105.0330	146.0598	+ H	1	0.990	5
leucine	11.2	236.1281	105.0333	86.0960	+ H	1	0.999	8
lysine	11	355.1652	105.0328	188.1072	+ H	2	0.979	9
methionie	9.4	252.0700	160.0753	146.0599	- H	1	0.978	7
MTA	1.7	314.0918	136.0616	97.0282	+ H (oxidizec	0	0.998	5
n-acetyl muramic acid	8.1	380.1338	105.0330	126.0543	+ H (water lc	1	0.982	7
n-acetyl-D-glycosamine	7.1	326.1234	105.0330	126.0539	+ H	1	0.956	9
ornithine	10.4	339.1350	120.0442	174.0911	- H	2	0.976	8
pantothenic acid	10.9	324.1442	184.0966	202.1063	+ H	1	0.992	9
phenylalanine	11.7	268.0979	120.0442	91.0507	- H	1	0.977	8
proline	7.9	220.0968	105.0330	70.0648	+ H	1	0.990	5
putrescine	11.2	297.1597	105.0330	176.1076	+ H	2	0.944	9
pyridoxine	7.3	274.1074	105.0330	256.0957	+ H	1	0.951	9
S-(1,2-dicarboxyethyl)glutathione	6.5	526.1142	143.0448	115.0023	- H	1	0.985	8
S-(5'-adenosyl)-L-homocysteine	3.4	505.1511	136.0611	97.0279	+ H (oxidizec	0	0.942	7
sarcosine	6.1	194.0812	105.0330	95.0487	+ H	1	0.990	5
serine	3.6	208.0615	134.0606	146.0595	- H	1	0.994	8
spermidine	8	354.2176	162.0911	105.0328	+ H	2	0.959	7
syringic acid	5.4	197.0456	123.0071	166.9982	- H	0	0.938	8
taurine	3.3	228.0336	106.9800	79.9564	- H	1	0.996	6
taurocholic acid	12	514.2844	N/A	N/A	- H	0	0.973	7
threonine	5.5	224.0917	105.0329	56.0491	+ H	1	0.998	5
thymidine	2.5	241.0830	125.0342	N/A	- H	0	0.980	6
tryptophan	11.4	307.1088	120.0442	134.0606	- H	1	0.999	5
tyrosine	8.4	284.0928	119.0492	120.0442	- H	1	0.999	5
valine	9.4	220.0979	176.1062	68.0495	- H	1	0.995	8
xanthine	6.9	255.0523	N/A	N/A	- H	1	0.975	8
xanthosine	8.5	387.0946	151.0247	193.0350	- H	1	0.978	6

Table S5. Significantly enriched transporter systems and carbohydrate active enzymes in the transcriptome of *R. pomeroyi* DSS-3 in co-culture with *T. pseudonana* . ** p < 0.01, * = p < 0.05, NS = not significant. GH = glycoside hydrolase, AA = auxiliary activity, CE = carbohydrate esterase. Alternating shading highlights multi-protein transporters.

SPO	Protein Tag	Fold Change	Adjusted p	Description
**Transporter Systems**				
SPO0591	WP_011046347.1	58.3	**	Dihydroxypropanesulfonate (DHPS) TRAP transporter, DctP
SPO0592	WP_011046348.1	43.5	**	Dihydroxypropanesulfonate (DHPS) TRAP transporter, DctQ
SPO0593	WP_011046349.1	37.9	**	Dihydroxypropanesulfonate (DHPS) TRAP transporter, DctM
SPO0608	WP_044027876.1	96.7	**	sugar ABC transporter, substrate binding protein
SPO0609	WP_011046365.1	60.7	**	sugar ABC transporter, ATP binding protein
SPO0610	WP_011046366.1	52.5	**	sugar ABC transporter, ATP binding protein
SPO0611	WP_011046367.1	50.3	**	sugar ABC transporter, permease
SPO0612	WP_011046368.1	46.4	**	sugar ABC transporter, permease
SPO0660	WP_011046411.1	3.6	**	*N* -acetyltaurine ABC transporter, substrate binding protein
SPO0661	WP_084791036.1	4.3	**	*N* -acetyltaurine ABC transporter, permease
SPO0662	WP_011046413.1	3.7	**	*N* -acetyltaurine ABC transporter, permease
SPO0663	WP_011046414.1	5.0	**	*N* -acetyltaurine ABC transporter, ATP-binding protein
SPO0664	WP_011046415.1	4.1	**	*N* -acetyltaurine ABC transporter, ATP-binding protein
SPO1017	WP_011046765.1	3.9	**	branched-chain amino acid ABC transporter, ATP binding protein
SPO1018	WP_011046766.1	4.3	**	branched-chain amino acid ABC transporter, ATP binding protein
SPO1019	WP_011046767.1	6.2	**	branched-chain amino acid ABC transporter, permease
SPO1020	WP_044027955.1	6.5	**	branched-chain amino acid ABC transporter, permease
SPO1021	WP_011046769.1	7.0	**	branched-chain amino acid ABC transporter, substrate binding protein
SPO1112	WP_011046859.1			TRAP transporter, DctP
SPO1113	WP_011046860.1	2.5	**	TRAP transporter, DctQ
SPO1114	WP_011046861.1	2.9	**	TRAP dicarboxylate transporter, DctM
SPO1485	WP_044028096.1	7.9	**	sodium:galactoside symporter family protein
SPO1490	WP_011047227.1	3.1	**	branched-chain amino acid ABC transporter, substrate binding protein
SPO1491	WP_011047228.1	3.1	*	branched-chain amino acid ABC transporter, permease
SPO1492	WP_011047229.1			branched-chain amino acid ABC transporter, permease
SPO1493	WP_011047230.1			branched-chain amino acid ABC transporter, ATP binding protein
SPO1707	WP_011047441.1	3.4	*	urea ABC transporter, ATP binding protein
SPO1707a	WP_011047442.1	3.5	**	urea ABC transporter, ATP binding protein
SPO1708	WP_011047443.1	4.2	**	urea ABC transporter, permease
SPO1709	WP_011047444.1	3.2	*	urea ATP transporter, permease
SPO1710	WP_044029188.1	4.3	**	urea ABC transporter, substrate binding protein
SPO1719	WP_011047455.1	3.7	**	TRAP dicarboxylate transporter, DctM (mannonate, putative)
SPO1720	WP_011047456.1	4.0	**	TRAP dicarboxylate transporter, DctQ
SPO1721	WP_044028157.1	3.1	**	TRAP dicarboxylate transporter, DctP
SPO1810	WP_011047543.1	4.8	**	sodium/solute symporter family protein (acetate, putative)
SPO1820	WP_011047553.1			sugar ABC transporter, substrate binding protein (glycerol-3-phosphate, putative)
SPO1821	WP_044028198.1	3.8	*	sugar ABC transporter, permease
SPO1822	WP_011047555.1	4.1	*	sugar ABC transporter, permease
SPO1823	WP_011047556.1			sugar ABC transporter, ATP binding protein
SPO1846	WP_011047579.1			branched-chain amino acid ABC transporter, substrate binding protein
SPO1848	WP_011047581.1	2.2	*	branched-chain amino acid ABC transporter, ATP binding protein
SPO1849	WP_011047582.1	2.3	**	branched-chain amino acid ABC transporter, ATP binding protein
SPO1850	WP_011047583.1	2.2	**	branched-chain amino acid ABC transporter, permease
SPO1851	WP_011047584.1	2.9	**	branched-chain amino acid ABC transporter, permease
SPO2530	WP_011048237.1			branched-chain amino acid ABC transporter, permease
SPO2531	WP_011048238.1			branched-chain amino acid ABC transporter, permease
SPO2532	WP_011048239.1			branched-chain amino acid ABC transporter, ATP binding protein
SPO2533	WP_011048240.1	4.6	**	branched-chain amino acid ABC transporter, ATP binding protein
SPO2534	WP_011048241.1			branched-chain amino acid ABC transporter, substrate binding protein
SPO2571	WP_011048276.1	3.2	**	TRAP transporter, DctM
SPO2572	WP_044028481.1	3.5	**	TRAP transporter, DctQ
SPO2573	WP_011048278.1	3.6	**	TRAP transporter, DctP
SPO2626	WP_011048328.1	2.9	**	TRAP transporter, DctM
SPO2627	WP_011048329.1	2.6	*	TRAP transporter, DctQ
SPO2628	WP_011048330.1	2.3	*	TRAP transporter, DctP
SPO2658	WP_011048360.1	47.8	**	cysteate ABC transporter, substrate binding protein
SPO2659	WP_011048361.1	51.4	**	cysteate ABC transporter, permease
SPO2660	WP_011048362.1	23.8	**	cysteate ABC transporter, permease

SPO2661	WP_011048363.1	22.2	**	cysteate ABC transporter, ATP binding protein
SPO2802	WP_011048502.1	2.9	**	sugar ABC transporter, substrate binding protein
SPO2803	WP_011048503.1	3.3	**	sugar ABC transporter, ATP binding protein
SPO2804	WP_011048504.1	5.2	**	sugar ABC transporter, permease
SPO2805	WP_044028562.1	6.3	**	sugar ABC transporter, permease
SPO3040	WP_011048734.1	157.6	**	polar amino acid ABC transporter, substrate binding protein (glutamine, putative)
SPO3041	WP_044028642.1	116.0	**	polar amino acid ABC transporter, permease
SPO3042	WP_011048736.1	52.4	**	polar amino acid ABC transporter, permease
SPO3043	WP_011048737.1	23.2	**	polar amino acid ABC transporter, ATP binding protein
SPO3290	WP_011048973.1	9.4	**	branched chain ABC transporter, ATP binding protein (phenylacetate, putative)
SPO3291	WP_011048974.1	4.7	**	branched chain ABC transporter, substrate binding protein
SPO3292	WP_011048975.1	6.8	**	branched chain ABC transporter, permease
SPO3294	WP_011048977.1	12.7	**	branched chain ABC transporter, permease
SPO3295	WP_044028707.1	16.5	**	branched chain ABC transporter, ATP binding protein
**CAZymes**				
SPO1844	WP_011047577.1	3.1	**	*N* -acetylglucosamine-6-phosphate deacetylase (CE9)
SPO3258	WP_011048948.1	2.8	**	glycosyl hydrolase 25 (GH25)
SPO0190	WP_011045959.1	2.7	**	oxidoreductase (AA3_2)
**DMSP Demethylation**				
SPO1913	WP_011047644.1	3.6	**	dimethylsulfoniopropionate demethylase
SPO1914	WP_011047645.1	3.6	**	acrylyl-CoA reductase

Table S6. Significantly enriched transporter systems and carbohydrate active enzymes in the transcriptome of *Stenotrophomonas* sp. SKA14 in co-culture with *T. pseudonana* and their location in PULs. ** p < 0.01, * = p < 0.05. GH = glycoside hydrolase, CBM = carbohydrate binding module, AA = auxiliary activity, CE = carbohydrate esterase, GT = glycosyl transferase.

Locus Tag	Protein Tag	Fold Change	Adjusted p	Description
**Transporter Systems**				
SSKA14_4109	WP_008268431.1	11.2	**	TonB Dependent Receptor (GluNac PUL)
SSKA14_4041	WP_008268362.1	8.7	**	NCS2 family permease: Xanthine/Uracil Family Permease
SSKA14_445	WP_008264850.1	8.3	**	TonB Dependent Receptor (Man PU)
SSKA14_174	WP_008264603.1	7.4	**	MFS transporter
SSKA14_3190	WP_040007328.1	6.7	**	TonB-dependent receptor, cobalamin
SSKA14_1710	WP_008266061.1	5.3	**	MFS transporter
SSKA14_2888	WP_008267272.1	5.0	**	sugar MFS transporter (GlcNac PUL)
SSKA14_2052	WP_008266402.1	4.3	**	MFS transporter of the (GalNac PUL)
SSKA14_3787	WP_008268127.1	4.0	**	amino acid permease
SSKA14_2708	WP_008267086.1	3.8	**	sugar MFS transporter (β-D-glucan PUL)
SSKA14_3866	WP_040009399.1	3.7	**	CitMHS family transporter, citrate transporter
SSKA14_993	WP_040009025.1	3.7	**	TonB-dependent vitamin B12 receptor
SSKA14_2346	WP_040009051.1	3.7	**	D-serine/D-alanine/glycine transporter
SSKA14_1433	WP_008265791.1	3.6	**	TonB-dependent receptor, cobalamin
SSKA14_1406	WP_006461445.1	3.5	**	arginine/agmatine antiporter
SSKA14_1199	WP_040008529.1	3.2	**	oligopeptide transporter, OPT family
SSKA14_103	WP_008264537.1	3.1	**	peptide MFS transporter
SSKA14_4238	WP_040007848.1	3.0	**	amino acid permease
SSKA14_3225	WP_008267572.1	2.7	**	hypothetical protein, possible xanthine/uracil family permease (IMG)
SSKA14_2695	WP_008267073.1	2.4	**	dicarboxylate/amino acid:cation symporter
SSKA14_2274	WP_004154263.1	2.3	**	nucleoside transporter NupC
SSKA14_2109	WP_008266462.1	2.2	**	proline/glycine betaine transporter ProP
SSKA14_582	WP_040006829.1	2.0	**	benzoate/H(+) symporter BenE family transporter
**CAZymes**				
SSKA14_1062	WP_008265435.1	9.8	**	DUF5110 domain-containing protein (GH31,CBM32,CBM51, GalNac PUL)
SSKA14_4140	WP_008268466.1	7.9	**	hypothetical protein (CBM32)
SSKA14_2691	WP_008267069.1	6.2	**	glycoside hydrolase family 92 protein (GH92)
SSKA14_1430	WP_040007852.1	5.3	**	glycoside hydrolase family 92 protein (GH92, Man PUL)
SSKA14_4417	WP_008268752.1	5.1	**	choline dehydrogenase (AA3)
SSKA14_2242	WP_040007085.1	4.8	**	family 20 glycosylhydrolase (GH20, GlcNac PUL)
SSKA14_158	WP_008264589.1	4.0	**	glycoside hydrolase family 2 protein (GH2, Man PUL)
SSKA14_1256	WP_008265632.1	3.1	**	glycoside hydrolase family 92 protein (GH92)
SSKA14_2811	WP_008267196.1	3.0	**	cellulose-binding protein (AA10,CBM73)
SSKA14_1958	WP_008266306.1	2.5	**	tetratricopeptide repeat protein (GT41)
SSKA14_133	WP_008264568.1	2.4	**	*N*-acetylglucosamine-6-phosphate deacetylase (CE9, GlcNac PUL)
SSKA14_150	WP_008264581.1	2.0	**	glycosyltransferase (GT2,GT4)
SSKA14_2339	WP_008266696.1	1.1		exo 1,3/1,4-beta-D-glucan glucohydrolase (EC 3.2.1.21)
**GlcNac PUL**				
SSKA14_2242	WP_040007085.1	4.8	**	beta-hexosaminidase (GH20)
SSKA14_4109	WP_008268431.1	11.2	**	TonB-dependent receptor
SSKA14_173	WP_008264602.1	12.4	**	glucokinase (EC 2.7.1.2)
SSKA14_2888	WP_008267272.1	5.0	**	sugar MFS transporter
SSKA14_413	WP_006391719.1			LacI family DNA-binding transcriptional regulator
SSKA14_1007	WP_008265386.1	2.0	*	glutamine-fructose-6-phosphate transaminase
SSKA14_133	WP_008264568.1	2.4	**	*N*-acetylglucosamine-6-phosphate deacetylase (CE9)
SSKA14_3769	WP_008268111.1			putative heparan-alpha-glucosaminide N-acetyltransferase
**Man PUL**				
SSKA14_158	WP_008264589.1	4.0	**	beta-mannosidase (EC 3.2.1.25) (GH2)
SSKA14_826	WP_008265210.1	2.0	*	N-acylglucosamine 2-epimerase
SSKA14_3154	WP_008267508.1			fructokinase
SSKA14_738	WP_008265126.1			L-fucose:H+ symporter permease
SSKA14_980	WP_008265359.1			transcriptional regulator, LacI family
SSKA14_1430	WP_040007852.1	5.3	**	alpha-1,2-mannosidase, putative subfamily (GH92)
SSKA14_445	WP_008264850.1	8.3	**	TonB-dependent receptor
SSKA14_2050	WP_008266400.1	4.8	**	TonB-dependent receptor
**GalNac PUL**				
SSKA14_2052	WP_008266402.1	4.3	**	MFS transporter (AgaP)
SSKA14_3302	WP_008267642.1	1.9	*	N-acetylglucosamine-6-phosphate deacetylase (CE9, AgaA)

SSKA14_1063	WP_008265436.1			transcriptional regulator, DeoR family (AgaR)
SSKA14_4056	WP_008268376.1	12.8	**	tagatose-bisphosphate aldolase noncatalytic subunit (AgaZ)
SSKA14_1072	WP_008265444.1			*N*-acetyl-D-glucosamine kinase (AgaK)
SSKA14_2667	WP_008267043.1			galactosamine 6-phosphate isomerase (AgaS)
SSKA14_1062	WP_008265435.1	9.8	**	(GH31,CBM32,CBM51)
SSKA14_802	WP_008265187.1	4.2	*	TonB-dependent receptor (Omp_Aga)
SSKA14_240	WP_040009289.1			Gfo/Idh/MocA family oxidoreductase (GH109)
**β-D-glucan PUL**				
SSKA14_1589	WP_008265935.1	2.4	*	sensor domain-containing diguanylate cyclase
SSKA14_1295	WP_008265669.1	1.4		cupin-like domain-containing protein
SSKA14_1667	WP_040006978.1	1.5		TonB-dependent receptor
SSKA14_2708	WP_008267086.1	3.8	**	sugar MFS transporter
SSKA14_1577	WP_040006979.1	1.4		LacI family DNA-binding transcriptional regulator
SSKA14_2339	WP_008266696.1	1.1		exo 1,3/1,4-beta-D-glucan glucohydrolase (EC 3.2.1.21)
SSKA14_1033	WP_008265408.1	1.3		hypothetical protein

Table S7. Significantly enriched transporter systems and carbohydrate active enzymes in the transcriptome of *Polaribacter dokdonensis MED152* in co-culture with *T. pseudonana* and their location in PULs. ** $p < 0.01$, * = $p < 0.05$. GH = glycoside hydrolase, CBM = carbohydrate binding module, AA = auxiliary activity, CE = carbohydrate esterase, GT = glycosyl transferase.

Locus Tag	Protein Tag	Fold Change	Adjusted p	Description
**Transporter Systems**				
MED152_00420	WP_015479862.1	10.1	**	TonB-dependent receptor, PUL2
MED152_05095	WP_015480785.1	6.0	**	MFS transporter, PUL3 (fucose)
MED152_05115	WP_015480789.1	3.3	**	MFS transporter, PUL3
MED152_05120	WP_015480790.1	6.7	**	MFS transporter, PUL3
MED152_05155	WP_015480797.1	4.9	**	TonB-dependent receptor, PUL3
MED152_06260	WP_015481012.1	2.3	**	peptide MFS transporter
MED152_06860	WP_015481128.1	2.8	**	Na+/proline symporter, PUL4
MED152_08060	WP_015481364.1	2.0	**	branched-chain amino acid transport system II carrier protein
MED152_08460	WP_015481441.1	4.0	**	sugar MFS transporter, PUL5
MED152_08475	WP_015481444.1	126.0	**	TonB-dependent receptor, PUL5
MED152_09830	WP_015481710.1	3.9	**	TonB-dependent receptor, PUL6
MED152_09865	WP_015481717.1	17.9	**	TonB-dependent receptor, PUL7
MED152_12804	WP_015482283.1	9.8	**	TonB-dependent receptor (cobalamin)
**CAZymes**				
MED152_11564	WP_015482037.1	9.2	**	family 16 glycosylhydrolase (GH16)
MED152_09780	WP_015481700.1	5.4	**	alginate lyase (PL6, PUL6)
MED152_00755	WP_015479928.1	4.5	**	T9SS C-terminal target domain-containing protein (GH81, CBM6)
MED152_07070	WP_051058582.1	4.3	**	glycoside hydrolase (GH113)
MED152_09875	WP_015481719.1	3.8	**	hypothetical protein (PL6)
MED152_09820	WP_015481708.1	3.3	**	heparinase (PL17, PUL6)
MED152_03735	WP_015480517.1	3.2	**	T9SS C-terminal target domain-containing protein (PL6)
MED152_09785	WP_015481701.1	3.0	**	polysaccharide lyase family 7 protein (PL7, PUL6)
MED152_08695	WP_015481488.1	2.5	**	glycosyl transferase family 2 (GT2)
MED152_03105	WP_041383822.1	2.2	**	response regulator (GT2)
MED152_05855	WP_015480934.1	2.2	**	glycogen synthase (GT4)
MED152_04045	WP_015480579.1	2.2	**	glycosyltransferase (GT2)
MED152_08490	WP_015481447.1	2.2	**	beta-N-acetylhexosaminidase (GH20, PUL5)
not assigned	WP_015480999.1	2.1	**	polysaccharide lyase family 7 protein (PL7)
**PUL2**				
MED152_00360	WP_015479851.1			LacI family transcriptional regulator
MED152_00365	WP_015479852.1			MFS transporter (maltose/moltooligosaccharide transporter)
MED152_00370	WP_015479853.1			beta-phosphoglucomutase
MED152_00375	WP_015479854.1			maltose phosphorylase (GH65)
MED152_00380	WP_015479855.1			sulfatase
MED152_00385	WP_015479856.1			alpha-amylase (GH13)
MED152_00390	WP_015479857.1			esterase
MED152_00395	WP_015479858.1			alpha-glucosidase/oligosaccharide 4-alpha-D-glucosyltransferase (GH31)
MED152_00400	WP_015479859.1			alpha-amylase (GH13)
MED152_00405	WP_015479860.1			alpha-amylase (GH13)
MED152_00410	WP_041383244.1			hypothetical protein
MED152_00415	WP_015479861.1			hypothetical protein (regulatory protein, LuxR family)
MED152_00420	WP_015479862.1	10.1	**	TonB-dependent receptor
MED152_00425	WP_041383746.1	7.3	**	RagB/SusD family nutrient uptake outer membrane protein
MED152_00430	WP_015479864.1	4.2	**	hypothetical protein
MED152_00435	WP_015479865.1	3.2	**	hypothetical protein
MED152_00440	WP_015479866.1			hypothetical protein (glycosyl hydrolase)
MED152_00445	WP_015479867.1			hypothetical protein (GH149)
MED152_00450	WP_015479868.1			glycosyl hydrolase family 17 (GH17)
MED152_00455	WP_015479869.1			glycosyl hydrolase family 30 (GH30)
MED152_00460	WP_015479870.1			MFS transporter (glycoside/pentoside/hexuronide:cation symporter, GPH family)
**PUL3**				
MED152_05050	WP_015480776.1			beta-glucosidase (GH3)
MED152_05055	WP_015480777.1			hypothetical protein
MED152_05060	WP_015480778.1			DNA polymerase III subunit epsilon
MED152_05065	WP_015480779.1			polyphosphate kinase
MED152_05070	WP_015480780.1			hypothetical protein
MED152_05075	WP_015480781.1	1.9	*	maltooligosyl trehalose synthase (GH13)
MED152_05080	WP_041383396.1	3.6	**	sugar kinase
MED152_05085	WP_015480783.1	2.0	*	solute:sodium symporter family transporter (putative myo-inositol cotransporter)
MED152_05090	WP_015480784.1			alpha-glucosidase (GH13)
MED152_05095	WP_015480785.1	6.0	**	MFS transporter (fucose permease)

MED152_05100	WP_015480786.1			glycoside hydrolase family 65 protein (GH65), trehalose and maltose hydrolase
MED152_05105	WP_015480787.1			mannan endo-1,4-beta-mannosidase (GH5)
MED152_05110	WP_015480788.1			trehalase (GH37)
MED152_05115	WP_015480789.1	3.3	**	MFS transporter (fucose permease)
MED152_05120	WP_015480790.1	6.7	**	MFS transporter (maltose/moltooligosaccharide transporter)
MED152_05125	WP_015480791.1			hypothetical protein
MED152_05130	WP_015480792.1			ASPIC-like protein
MED152_05135	WP_015480793.1			ASPIC-like protein
MED152_05140	WP_015480794.1			hypothetical protein
MED152_05145	WP_015480795.1			ASPIC-like protein
MED152_05150	WP_015480796.1			RagB/SusD family nutrient uptake outer membrane protein
MED152_05155	WP_015480797.1	4.9	**	SusC/RagA family TonB-linked outer membrane protein
MED152_05160	WP_015480798.1			DUF1080 domain-containing protein
MED152_05165	WP_015480799.1			sugar phosphate isomerase/epimerase
MED152_05170	WP_015480800.1			gfo/Idh/MocA family oxidoreductase
MED152_05175	WP_015480801.1			nucleoside symporter
MED152_05180	WP_015480802.1	2.2	**	sugar phosphate isomerase/epimerase
MED152_05185	WP_015480803.1	2.2	**	AraC family transcriptional regulator
MED152_05190	WP_015480804.1	2.4	**	oxidoreductase (AA3)
MED152_05195	WP_015480805.1			gluconate 2-dehydrogenase subunit 3 family protein
**PUL4**				
MED152_06840	WP_015481124.1			hypothetical protein
MED152_06845	WP_015481125.1			hypothetical protein
MED152_06850	WP_015481126.1			SusC/RagA family TonB-linked outer membrane protein
MED152_06855	WP_015481127.1			SusD/RagB family nutrient-binding outer membrane lipoprotein
MED152_06860	WP_015481128.1	2.8	**	sodium:solute symporter "proline symporter"
MED152_06865	WP_015481129.1			diadenosine tetraphosphatase
MED152_06870	WP_015481130.1			rhodanese-like domain-containing protein
MED152_06875	WP_015481131.1			hypothetical protein
MED152_06880	WP_015481132.1			purine-nucleoside phosphorylase
MED152_06885	WP_015481133.1			DUF1679 domain-containing protein
**PUL5**				
MED152_08455	WP_015481440.1			aldose 1-epimerase
MED152_08460	WP_015481441.1	4.0	**	sugar MFS transporter
MED152_08465	WP_015481442.1	3.7	**	DNA-binding response regulator
MED152_08470	WP_015481443.1	9.1	**	histidine kinase
MED152_08475	WP_015481444.1	14.0	**	SusC/RagA family TonB-linked outer membrane protein
MED152_08480	WP_015481445.1	9.4	**	SusD/RagB family nutrient-binding outer membrane lipoprotein
MED152_08485	WP_015481446.1	6.7	**	glucosamine-6-phosphate deaminase
MED152_08490	WP_015481447.1	2.3	**	beta-N-acetylhexosaminidase (GH20)
MED152_08495	WP_015481448.1			N-acetylglucosamine kinase
MED152_08500	WP_015481449.1			hypothetical protein
**PUL6 and 7**				
MED152_09760	WP_015481697.1			adenosylhomocysteinase
MED152_09765	WP_015481698.1	9.2	**	PepSY domain-containing protein, Uncharacterized iron-regulated membrane protein
MED152_09770	WP_015481699.1			sodium:sulfate symporter
MED152_09780	WP_015481700.1	5.4	**	poly(beta-D-mannuronate) lyase (PL6)
MED152_09785	WP_015481701.1	3.0	**	polysaccharide lyase family 7 protein (PL7)
MED152_09790	WP_015481702.1			gluconokinase
MED152_09795	WP_015481703.1			6-phosphogluconate dehydrogenase
MED152_09800	WP_015481704.1	2.9	**	putative Mn2+ Fe2+ transporter
MED152_09805	WP_015481705.1			polysaccharide lyase family 7 protein (PL7)
MED152_09810	WP_015481706.1			3-oxoacyl-ACP reductase FabG
MED152_09815	WP_015481707.1	14.5	**	Por secretion system C-terminal sorting domain-containing protein
MED152_09820	WP_015481708.1	3.3	**	heparinase (PL17)
MED152_09825	WP_015481709.1			cupin domain-containing protein
MED152_09830	WP_015481710.1	3.9	**	TonB-dependent receptor
MED152_09835	WP_015481711.1	2.0	*	RagB/SusD family nutrient uptake outer membrane protein
MED152_09840	WP_015481712.1			hypothetical protein
MED152_09845	WP_015481713.1			PKD domain-containing protein
MED152_09850	WP_015481714.1			FadR family transcriptional regulator
MED152_09855	WP_015481715.1			MFS transporter
MED152_09860	WP_015481716.1			Short-chain dehydrogenase/reductase
MED152_09865	WP_015481717.1	17.9	**	TonB-dependent receptor
MED152_09870	WP_015481718.1	7.8	**	RagB/SusD family nutrient uptake outer membrane protein
MED152_09875	WP_015481719.1	3.8	**	poly(beta-D-mannuronate) lyase (PL6)
MED152_09880	WP_083903258.1			ATP-binding protein
MED152_09885	WP_041383578.1	2.1	**	hypothetical protein
